# Location Specificity of Transcranial Electrical Stimulation on Neuronal Electrodynamics: A Mathematical Model of Ion Channel Gating Dynamics and Ionic Flux Due to Neurostimulation

**DOI:** 10.3389/fncom.2019.00017

**Published:** 2019-04-04

**Authors:** Kaia R. Lindberg, Edward T. Dougherty

**Affiliations:** Mathematics Department, Roger Williams University, Bristol, RI, United States

**Keywords:** location specificity of neuronal electrodynamics, neurostimulation induced calcium flux, mathematical model, transcranial electrical stimulation model, finite element method simulation

## Abstract

Transcranial Electrical Stimulation (TES) continues to demonstrate success as a medical intervention for individuals with neurodegenerative diseases. Despite promising results from these neuromodulation modalities, the cellular level mechanisms by which this neurotherapy operates are not fully comprehended. In particular, the effects of TES on ion channel gating and ion transport are not known. Using the Poisson-Nernst-Planck model of electrodiffusion, coupled with a Hodgkin-Huxley based model of cellular ion transport, we present a model of TES that, for the first time, integrates electric potential energy, individualized ion species, voltage-gated ion channels, and transmembrane ionic flux during TES administration. Computational simulations are executed on a biologically-inspired domain with medically-based TES treatment parameters and quantify neuron-level electrical processes resulting from this form of neurostimulation. Results confirm prior findings that show that TES polarizes the cell membrane, however, these are extended as simulations in this paper show that polarization occurs in a location specific manner, where the type and degree of polarization depends on the position on the membrane within a node of Ranvier. In addition, results demonstrate that TES causes ion channel gating variables to change in a location specific fashion and, as a result, transmembrane current from distinct ion species depends on both time and membrane location. Another simulation finding is that intracellular calcium concentrations increase significantly due to a TES-induced calcium influx. As cytosolic calcium is critical in intracellular signaling pathways that govern proper neurotransmitter secretion as well as support cell viability, this alteration in calcium homeostasis suggests a possible mechanism by which TES operates at the neuronal level to achieve neurotherapeutic success.

## 1. Introduction

Transcranial electrical stimulation (TES) is a group of neurostimulation therapies that deliver low doses of electric current to targeted brain regions via noninvasive electrodes placed on a patient's scalp. The most common type of TES is transcranial direct current stimulation (tDCS), which administers a constant amount of electrical energy during therapy sessions. Other forms of TES include transcranial alternating current stimulation (tACS) as well as transcranial random noise stimulation (tRNS), both of which utilize a non-constant dosage of electric current (Paulus, 2011; Antal and Paulus, 2013). Most recently, high-definition TES has been introduced as a neurostimulation approach that achieves a more focused delivery of electrical energy through the use of numerous smaller anode and cathode electrodes, as opposed to just the single larger-sized anode and cathode traditionally used in tDCS, tACS, and tRNS (Borckardt et al., 2012; Caparelli-Daquer et al., 2012).

Clinical experiments clearly show that TES is an effective intervention for treating conditions that manifest from neurodegenerative disorders. Parkinson's disease patients, for example, have demonstrated enhanced movement capabilities and memory skills from TES (Boggio et al., 2006; Johnson et al., 2008). Also, individuals suffering from Alzheimer's disease have demonstrated improved recognition and memory capabilities (Boggio et al., 2009, 2011). Further, TES has shown to improve language re-learning in dementia patients (Wang et al., 2013; Yun et al., 2016). In addition to clinical findings, biological experiments have begun to show the effects of TES on membrane polarization (Liebetanz et al., 2002; Bikson et al., 2004; Stagg and Nitsche, 2011; Das et al., 2016) and calcium homeostasis (Islam et al., 1995; Nitsche et al., 2003; Adams et al., 2017), however difficulties in capturing ion channel state, ionic flux, and intracellular calcium concentrations continuously over time with a high sampling frequency yields limited neurostimulation data at the cellular level (Adams et al., 2017). Thus, the direct influence of an applied TES electric current on voltage-gated ion channel states as well as other cellular level mechanism by which TES operates is largely unknown (Nitsche et al., 2008).

In partnership with biomedical research, mathematical modeling and computational simulation have helped to enhance the neurological communities' understanding of TES. Recent models have begun to describe the impact of electrical stimulation on electric potential around neural tissue (Mandonnet and Pantz, 2011). In addition, biodomain models have provided a means to begin to characterize the influence of electrical energy on transmembrane potential using volume averaging approaches (Sadleir, 2010; Dougherty et al., 2014). These models support the physiological conclusion that TES influences the neuron by slightly polarizing the cell membrane (Nitsche et al., 2008), however, the level of biological abstraction of their mathematical formulations inherently prohibits a quantitative description of individual ion species and their movements around and through the neuron cell wall. A mathematical model of TES that incorporates the electrodiffusion of distinct ion types throughout the intracellular and extracellular domains, as well as their mobility across the cell membrane via voltage-gated ion channels, would for the first time give researchers the capability to computationally assess the impact that a TES-based electric field has on ion channel gating and subsequent ionic flux.

In this paper, we present a novel mathematical model of TES that provides a description of its effects on cellular level neuronal electrodynamics. The model integrates the Poisson-Nernst-Planck electrodiffusion system of partial differential equations (PDEs) and Hodgkin-Huxley motivated boundary conditions for cell membrane ionic flux with extracellular boundary conditions that model TES treatments. Four ion species, namely sodium, potassium, chloride, and calcium, are incorporated in the model. We include calcium in this model as cytosolic calcium is known to be an essential member of the intracellular biochemical network that triggers proper neurotransmitter secretion, and in addition, holds an integral connection with neurodegenerative diseases (Bezprozvanny, 2009; Marambaud et al., 2009; Calì et al., 2014; Surmeier et al., 2017). The TES model is then simulated on a biologically-inspired computational domain (Sosinsky et al., 2005; Chang and Rasband, 2013; Arancibia-Cárcamo et al., 2017) that includes intracellular, extracellular, and membrane regions. Using *in silico* experiments, we examine the impact of TES on (i) extracellular and intracellular electric potential, (ii) resting membrane potential along the node of Ranvier, (iii) voltage-dependent ion channel gating, (iv) ionic membrane flux, and (v) extracellular and intracellular ion diffusion.

Results demonstrate that a simulated TES current does in fact instantaneously polarize the transmembrane resting potential, however, it does so in a location-dependent manner, where depolarization occurs in a portion of the node of Ranvier and hyperpolarization in other regions. In turn, there is a location-dependent effect on voltage-gated ion channel states, which directly impacts ion channel permeability. Additionally, results show a location-dependent influence on ion membrane flux, with regions along the membrane that exhibit significant increases in sodium and calcium intracellular influx. Of particular importance to applications focusing on neurodegenerative diseases, simulations of TES show calcium intracellular concentrations can increase by up to 71.65% along some regions of the node of Ranvier. In addition, the total calcium concentration in the intracellular domain increases by 63.86% due to TES.

To our knowledge, this paper presents the first model of TES that incorporates neurophysiology with individual ion species and transmembrane ionic fluxes. We hope that the models, simulations, and results presented in this work help expand the research communities' understanding of the neurological mechanisms by which TES operates, and in addition, broadens the utility of mathematical modeling and simulation for computational neuroscience research.

## 2. Materials and Methods

### 2.1. Poisson-Nernst-Planck Model

The time-dependent Poisson-Nernst-Planck (PNP) system of partial differential equations (PDEs) can be used to model ion electrodiffusion around and within a neuron (Horng et al., 2012; Dione et al., 2016). The Nernst-Planck equation, which describes particle movement due to both diffusion and electrostatic forces, is given by

(1) $$\begin{array}{l} \frac{\partial n_{i}}{\partial t}+\nabla \cdot F_{i}=0, \end{array}$$

where the ion flux, *F*_*i*_, is given by

(2) $$\begin{array}{l} F_{i}=-D_{i}\left(\nabla n_{i}+\frac{n_{i}}{\alpha_{i}}\nabla \phi \right), \end{array}$$

where $n_{i}=n_{i}\left(\vec{x},t\right)$ and $\phi =\phi \left(\vec{x},t\right)$ represent the concentration of the *ith* ion and the electric potential energy, respectively, both of which are unknown quantities to be solved for. In addition, constant *D*_*i*_ is the diffusivity in water for the *ith* ion, and the constant α_*i*_ equals $\frac{RT}{Fz_{i}}$, where *R*, *T*, and *F* are the gas constant, temperature of the medium, and Faraday's constant, respectively.

The Poisson equation portion of the PNP system quantifies the electric potential energy due to ion concentrations and their relative valences, and is given by

(3) $$\begin{array}{l} \nabla \cdot \left(\epsilon \nabla \phi \right)=-F\underset{i=1}{\sum }z_{i}n_{i}, \end{array}$$

where *z*_*i*_ is the valence of ion *i*. In addition, ϵ denotes the permittivity of the medium, equaling ϵ_*c*_ · ϵ_0_ in intracellular and extracellular regions, and ϵ_*memb*_ · ϵ_0_ in the cell membrane. Here, ϵ_0_ is given by vacuum permittivity while ϵ_*c*_ and ϵ_*memb*_ are relative permittivities of the intra/extra-cellular and membrane domains, respectively.

In this paper, four ion species are used in the PNP model, namely sodium (*Na*^+^), potassium (*K*^+^), calcium (*Ca*^+2^), and chloride (*Cl*^−^); thus, equation 1 is realized four times, and the summation term of equation 3 contains four terms.

### 2.2. Computational Domain

The model is simulated on a biologically-inspired two-dimensional domain representing a portion of a neuron axon that includes a single node of Ranvier, the neuronal region rich in ion channels and transmembrane ionic transport. The domain was constructed using both the myelinated and unmyelinated regions of the membrane, and biologically accurate dimensions were incorporated (Sosinsky et al., 2005; Lopreore et al., 2008; Briegel et al., 2009; Chang and Rasband, 2013; Pods et al., 2013; Maxwell, 2014; Dione et al., 2016; Arancibia-Cárcamo et al., 2017; Rogers and Team of Encyclopedia, 2018). The three subregions of the computational domain consists of (i) intracellular space, (ii) membrane, (iii) and extracellular space.

**Figure 1:**
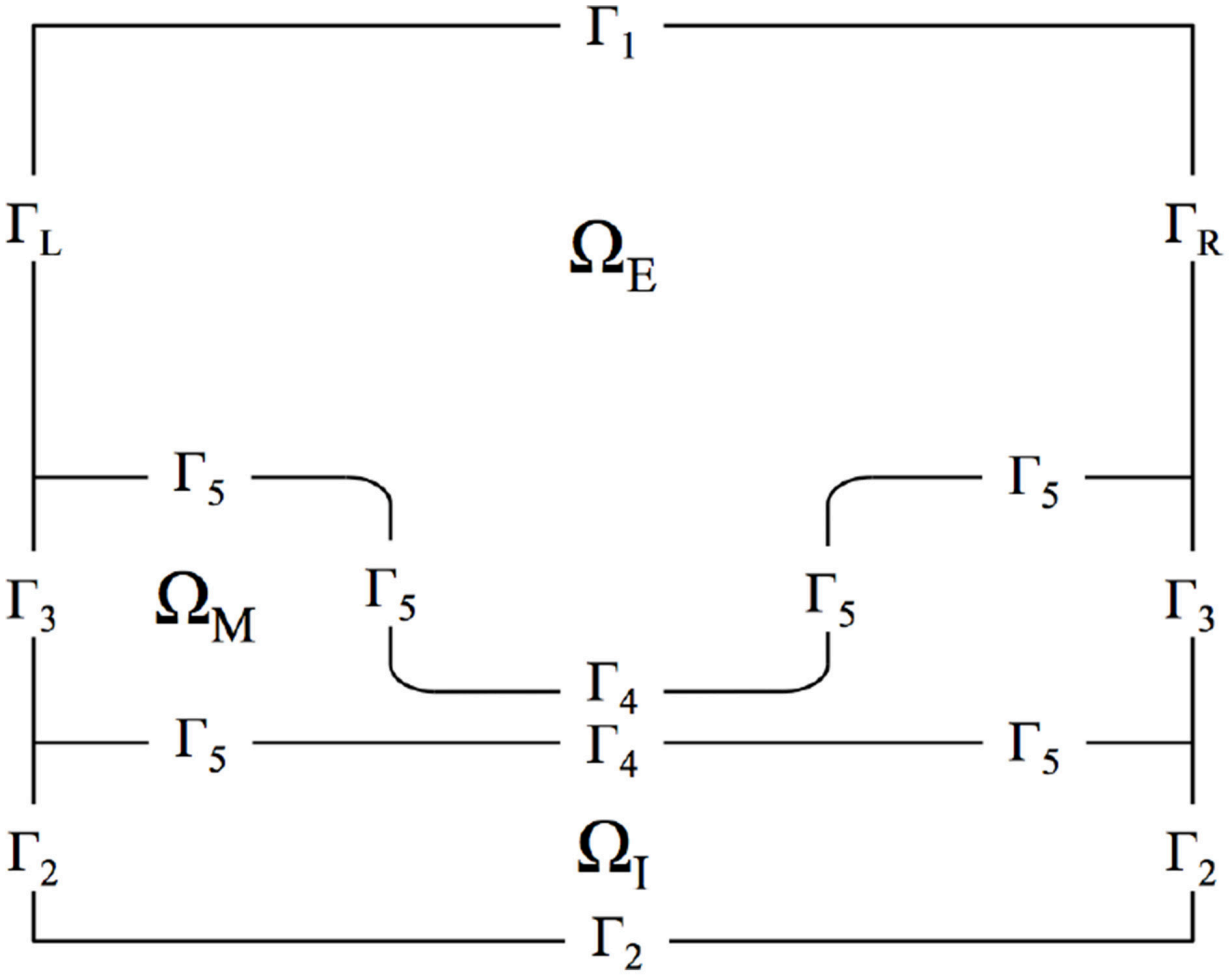
Diagram of computational domain with intracellular (Ω_*I*_), membrane (Ω_*M*_), and extracellular (Ω_*E*_) subdomains. The diagram also includes labels for each boundary in the domain. Γ_*L*_ and Γ_*R*_ are the boundaries for the left and right sides of the extracellular space, respectively. Γ_1_ is the boundary for the top of the extracellular space and Γ_2_ labels the exterior boundaries for the intracellular subdomain. Γ_3_ is the exterior boundary of the membrane and Γ_5_ labels the boundary between the membrane and intra/extra-cellular space other than in the node of Ranvier, which is labeled by Γ_4_.

Figure 1 presents the domain, noting the locations of the three regions as well as all domain boundaries. The length of the axon portion of the domain is 4 μm (Lopreore et al., 2008; Dione et al., 2016) with the nodal portion having a length of 1 μm (Sosinsky et al., 2005; Lopreore et al., 2008; Dione et al., 2016; Arancibia-Cárcamo et al., 2017; Rogers and Team of Encyclopedia, 2018). The radius of the myelinated and unmyelinated sections of the membrane are 0.406 μm (Lopreore et al., 2008; Dione et al., 2016) and 0.005 μm (Briegel et al., 2009; Chang and Rasband, 2013; Pods et al., 2013), respectively. The radius of the intracellular space is 0.434 μm (Lopreore et al., 2008; Dione et al., 2016), and the whole domain, i.e., intracellular, membrane, and extracellular spaces, has a radius of 2 μm (Lopreore et al., 2008; Dione et al., 2016).

**Figure 2:**
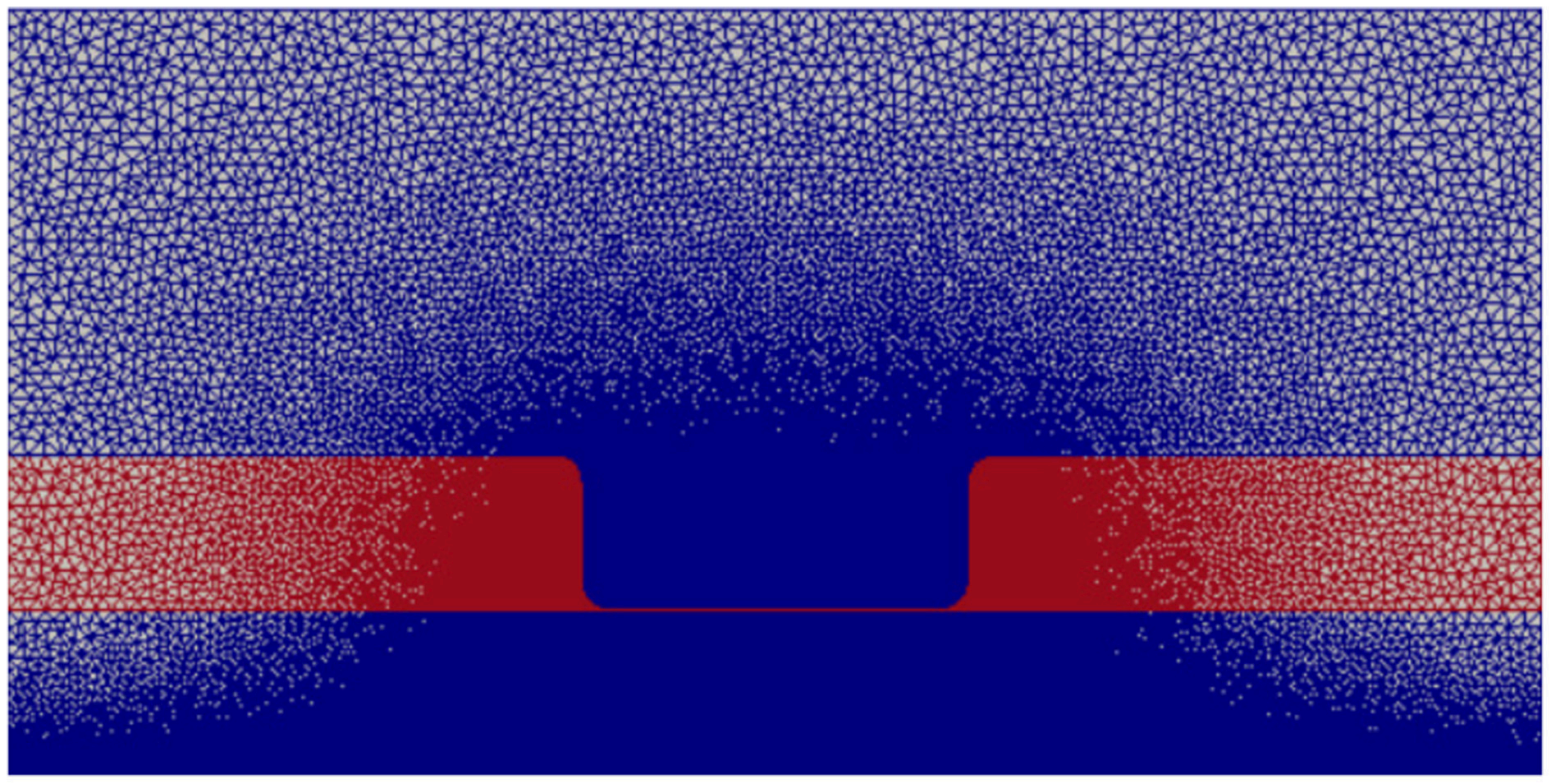
Computational mesh with nodes on which the PDEs are solved. Intracellular and extracellular subdomains are shown in blue and the membrane region is shown in red.

Figure 2 displays the discretized computational mesh used in each simulation; in this mesh, there are 725,528 elements, with 67,810 nodes in the membrane, 502,644 in the intracellular space, and 159,410 in the extracellular space. The mesh has a much finer grid resolution in the Debye layer, the extracellular space directly adjacent to the membrane, as well as its neighboring intracellular space; this finer discretization is necessary to accurately model the rapid solution changes that take place in these regions of the domain (Pods et al., 2013).

### 2.3. Boundary Conditions

Equation (1) is defined on the intracellular and extracellular regions of the domain, namely Ω_*I*_ ∪ Ω_*E*_, whereas Equation (3) is defined on the entire domain Ω = Ω_*I*_ ∪ Ω_*M*_ ∪ Ω_*E*_ (Pods et al., 2013). Thus, boundary conditions for these equations must be stipulated on these respective boundaries. In addition, to appropriately model TES at the cellular level, boundary conditions for the Nernst-Planck equation and the Poisson equation must be specified to model TES administration as well as ion transport across the cell membrane. These conditions are described in the following sections.

#### 2.3.1. TES Boundary Conditions

On the extracellular space top boundary (Γ_1_), the concentrations of each ion are set to a constant bulk solution value using the non-homogeneous Dirichlet boundary condition

(4) $$\begin{array}{l} n_{i}=n_{i}^{0},\;\vec{x}\in \Gamma_{1}. \end{array}$$

In this work, we focus on a constant stimulation source, i.e., tDCS, and the next two boundary conditions achieve this form of TES. The electric potential on right side of the extracellular space (Γ_*R*_) is maintained at a value of zero using the homogeneous Dirichlet boundary condition

(5) $$\begin{array}{l} \phi =0,\;\vec{x}\in \Gamma_{R}. \end{array}$$

For the first 2 ms of the simulation, the electric potential on the left side of the extracellular space (Γ_*L*_) is set to zero; after this time, TES is simulated by changing this value to 0.1 V (Faria et al., 2011; Gasca et al., 2011; Datta et al., 2013). This TES administration is represented with the time-dependent Dirichlet boundary condition

(6) $$\begin{array}{l} \phi =\{\begin{array}{cc} 0 & :t\leq 2\text{ms},\vec{x}\in \Gamma_{L}\\ 0.1 & :t>2\text{ms},\vec{x}\in \Gamma_{L} \end{array} \end{array}$$

Note that alternative forms of TES can be simulated simply by implementing a non-constant value of ϕ once stimulation is activated in Equation (6).

For Equation (1), on all boundaries except the membrane, ion flux is set to zero:

(7) $$\begin{array}{l} F_{i}\cdot \vec{n}=0,\vec{x}\in \Gamma_{2}\cup \Gamma_{3}\cup \Gamma_{5}\cup \Gamma_{L}\cup \Gamma_{R}, \end{array}$$

and the charge density flux for Equation (3) is set to zero on all the boundaries not governed by the TES source or ground using the homogeneous Neumann condition

(8) $$\begin{array}{l} \epsilon \nabla \phi \cdot \vec{n}=0,\vec{x}\in \Gamma_{1}\cup \Gamma_{2}\cup \Gamma_{3}. \end{array}$$

#### 2.3.2. Hodgkin-Huxley Gating Equations

The transport of ions across the membrane wall within the node of Ranvier is governed by the non-homogeneous Neumann boundary condition

(9) $$\begin{array}{l} F_{i}\cdot \vec{n}=f_{i}^{memb}\left(n_{i},\phi ,t\right),\vec{x}\in \Gamma_{4}, \end{array}$$

where $f_{i}^{memb}$ is a time and position dependent function that incorporates a Hodgkin-Huxley based model (Hodgkin and Huxley, 1952; Kay and Wong, 1987; Tuckwell, 2012) to quantify sodium, potassium, chloride, and calcium ion flux (see Appendix B). The membrane flux $f_{i}^{memb}$ uses the transmembrane voltage *V* = ϕ_*I*_ - ϕ_*E*_ in its calculation, which is computed at every point along the membrane in the discretized mesh (Dione et al., 2016).

### 2.4. Numerical Implementation

Equations (1) and (3) are decoupled using the Gauss-Seidal method (Sundnes et al., 2006). The solution algorithm consists of the following steps:

1. Solve equation 3 for ϕ at time step *k* + 1 given ion concentrations at time step *k*, $n_{i}^{k}$, with boundary conditions given by Equations (5), (6), and (8). Let ϕ^*k* + 1^ denote this solution.
2. Solve for $f_{i}^{memb}$ given $n_{i}^{k}$ and ϕ^*k* + 1^ (see section 2.3.2).
3. Solve Equation (1) for *n*_*i*_, for each ion type, at time step *k* + 1 given ϕ^*k* + 1^, with boundary conditions given by Equations (4), (7), and (9). Let $n_{i}^{k+1}$ denote these solutions.

The result is numerical solutions of ϕ and *n*_*i*_ at time step *k* + 1. This iterative sequence is initiated using prescribed intracellular and extracellular initial concentrations of each ion type, and is repeated until the end of the simulation. Within this loop, an inner iteration is used in step 2 to solve the Hodgkin-Huxley system with a smaller time step. This approach ensures the accuracy of the ion flux at the membrane and enables a larger time step for the more computationally intensive PDE solvers in steps 1 and 3. Given that the transmembrane voltage and subsequent flux vary along the node of Ranvier, a different realization of these ordinary differential equations (ODEs) is needed to be solved at every point along the membrane. In this work, the discretized domain generates 1,700 nodes along the membrane, thus the Hodgkin-Huxley ODE system was instantiated and solved for 1,700 times at each simulation time step.

The PDE in step 1 is solved using the finite element method. The PDE system in step 3 is discretized in time using the θ-rule and space using the finite element method (Mardal et al., 2003). The value of θ was set equal to 1, which corresponds to the Backward Euler method, due to its L-stability properties (Hairer and Wanner, 1996). Resulting weak formulations for these equations are presented in Appendix A. The Hodgkin-Huxley ODEs are solved using LSODE (Hindmarsh, 1983; Hairer and Wanner, 1996). This iterative implementation approach enables numerical solvers tailored to each individual equation to be used (Langtangen and Tveito, 2003), as well as individualized time steps for the PDEs and ODEs.

### 2.5. Computational Tools

The computational domain (Figure 2) was constructed and discretized using GMSH (Geuzaine and Remacle, 2009). The FEniCS computing platform (Alnæs et al., 2015) was used to solve the partial differential equations. This Python based library offers packages to solve finite element weak formulations subject to all boundary and initial conditions. In addition, Python's SciPy library was used to access the LSODE method (Jones et al., 2001).

Given the complexity of the mathematical model and solution approach, an object-oriented implementation of the code was developed. This approach compartmentalizes major modeling components into “classes,” and in doing so, facilitates debugging as each class can be analyzed independently, and in addition, improves code readability. Furthermore, while object-oriented implementations often take more time to design and implement than traditional procedural implementations, a significant advantage of using a class-based structure is its inherent ability to support alternative applications. For example, changes in domain geometry, mesh resolution, TES parameters, or even in the set of ions used can be effortlessly incorporated with virtually no changes to the software (Dougherty and Turner, 2016).

A class for the Nernst-Planck equation incorporates all information needed to solve this equation. This includes its associated weak formulation, diffusivity values, boundary conditions, time steps, and domain information. There are eight instantiations of this class, one for each ion type for both the intracellular and extracellular domains. A separate class is used to solve for $f_{i}^{memb}$ needed in step 2 of the iterative solution algorithm. There is an instantiation of this class for each of the four ion types. These membrane current classes in turn possess an object dedicated to solving the Hodgkin-Huxley differential equations, which generates solutions for the gating variables *m, n*, and *h* (see section 2.3.2). There are 1,700 instantiations of this class, one for each discretized point on the membrane. Information in this Hodgkin-Huxley class is used by the membrane current class to resolve $f_{i}^{memb}$ along the membrane, which is then used by the Nernst-Planck class via access to the membrane current class.

### 2.6. Numerical Simulations

A 20 ms simulation of TES was performed via the boundary condition given by Equation (6). A time step of 0.01 ms was selected for the outer iteration of the solution algorithm (section 2.4) as this value is small enough to accurately model the changes in electric potential and ion concentrations (Pods et al., 2013). For solving the inner iteration of step 2, the ODE system was solved with a maximal time step of 0.0005 ms. As described by Equation (6), TES is simulated by changing the Dirichlet boundary condition value from 0 V to 0.1 V on the left boundary of the extracellular space after *t* = 2 ms, which was selected as this allows concentration gradients and transmembrane ionic flux to achieve equilibrium; this stimulation dosage is consistent with electric potentials achieved during TES sessions (Fregni et al., 2006; Miranda et al., 2006; Datta et al., 2013). This allows the electric potential, transmembrane voltage, ion channel gating variables, ionic flux, and ion concentrations before and after electrical stimulation to be directly compared, thus enabling a direct assessment of the specific impact of TES on neuronal electrodynamics. All simulation parameter values are presented in Table 1, and all values used in the model and simulations are taken from published biomedical literature and previous neuronal mathematical models (Lopreore et al., 2008; Pods et al., 2013; Dione et al., 2016). Simulation run time was approximately 3 days, 18 min, and 9 seconds, on a computer using a fourth generation Intel XEON processor with 3.7 GHz and eight cores.

**Table 1:** Simulation parameters.

**Parameter**	**Value**
Perfect gas constant	8.31454 $\frac{\text{J}}{\text{mole}\cdot \text{K}}$
Faraday's constant	96485 $\frac{\text{C}}{\text{mole}}$
Temperature	279.450 K
Vacuum permittivity	$\text{8.88542}\cdot {\text{10}}^{-12}\frac{\text{C}}{\text{m}\cdot \text{V}}$
Cytosol relative permittivity	80
Membrane relative permittivity	2
Initial *Na*^+^ intracellular concentration	12 mM
Initial *Na*^+^ extracellular concentration	145 mM
Initial *K*^+^ intracellular concentration	155 mM
Initial *K*^+^ extracellular concentration	4 mM
Initial *Ca*^+2^ intracellular concentration	0.0001 mM
Initial *Ca*^+2^ extracellular concentration	1 mM
Initial *Cl*^−^ intracellular concentration	166.8 mM
Initial *Cl*^−^ extracellular concentration	123.27 mM
*Na*^+^ Diffusivity	$\text{1.33}\cdot {\text{10}}^{-9}\frac{{\text{m}}^{\text{2}}}{\text{s}}$
*K*^+^ Diffusivity	$\text{1.96}\cdot {\text{10}}^{-9}\frac{{\text{m}}^{\text{2}}}{\text{s}}$
*Ca*^+2^ Diffusivity	$\text{0.5}\cdot {\text{10}}^{-9}\frac{{\text{m}}^{\text{2}}}{\text{s}}$
*Cl*^−^ Diffusivity	$\text{2.0}\cdot {\text{10}}^{-9}\frac{{\text{m}}^{\text{2}}}{\text{s}}$
Time step	0.01 ms
Hodgkin-Huxley time step	0.0005 ms
Simulation start	0 ms
Time of TES application	2 ms
Total simulation time	20 ms

An iterative implementation and testing approach was used to verify the accuracy of the model implementation. First, individual solvers for the PDEs given by Equation (1) and (3) were constructed and validated against the online PDE solver DiffpackSE (Bruaset and Langtangen, 1997; Langtangen, 2003). Second, the Hodgkin-Huxley ODE model was implemented and verified independently of the PDEs, thus ensuring that changes in intracellular and extracellular electric potential and ion concentrations at the membrane correctly compute gating variable states as well as flux during membrane polarization (Hodgkin and Huxley, 1952; Kay and Wong, 1987; Tuckwell, 2012). Third, these three solvers were integrated into a single solution code using the object-oriented approach as detailed in section 2.5. Fourth, verification of the complete code came by comparing sodium and potassium membrane flux time courses and magnitudes to results from previous PNP modeling implementations (Lopreore et al., 2008; Pods et al., 2013; Dione et al., 2016). Fifth, the transmembrane voltages, intra/extra-cellular ion concentrations, ion channel gating variables, and membrane current fluxes predicted by the complete, fully-coupled model were compared to the isolated Hodgkin-Huxley code (see section 2.5) to validate the accuracy of the fully integrated, coupled implementation used in all simulations. Finally, we draw comparisons between results of the model and those of published medical studies and biological experiments when available.

## 3. Results

### 3.1. Transmembrane Voltage Polarization Exhibits Location Specificity

**Figure 3:**
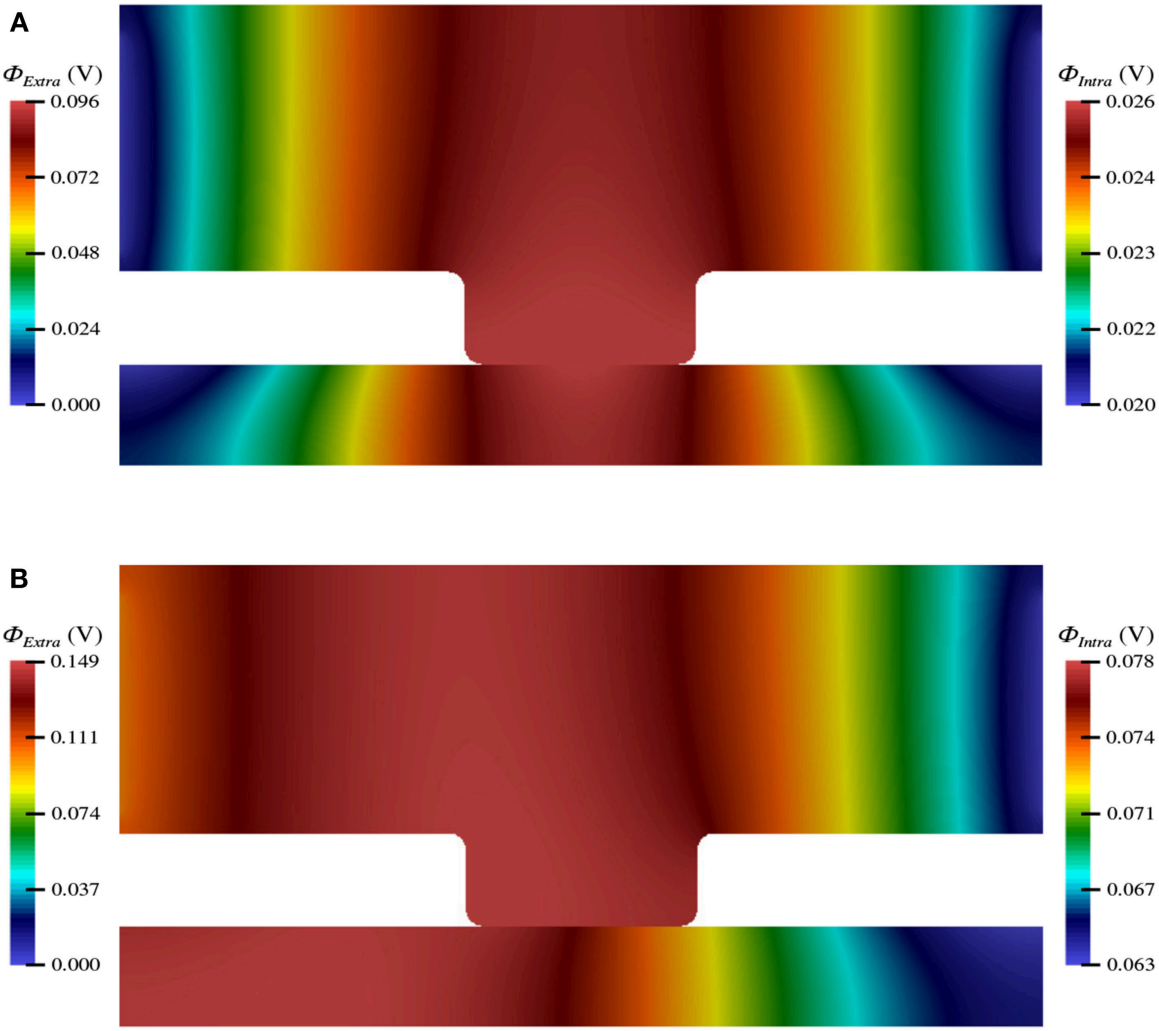
Electric potential energy (ϕ) throughout the computational domain at *t* = 0 ms **(A)** and *t* = 20 ms **(B)**.

The electric potential energy, ϕ, throughout the neuronal domain at both the beginning and the end of the simulation is shown in Figure 3. Here, changes in both the distribution and magnitude of ϕ from TES are observed. In particular, prior to neurostimulation application, the electric potential distribution is highly symmetric (Figure 3A), however, after TES administration, the domain is highly asymmetric; the majority of high voltage areas are concentrated on the left side of the domain, juxtaposed with the stimulation source boundary condition ϕ = 0.1 V, and electric potential declines more rapidly as the ground boundary is approached (Figure 3B). In addition, the maximum extracellular electric potential value increases by 55.2% from 0.096 V at the start of the simulation to 0.149 V at the end, which due to ionic electrodiffusion, is 49.0% greater than the anode source voltage of 0.1 V. Further, intracellular values for ϕ increase themselves from a minimum and maximum of 0.020 V and 0.026 V to 0.063 V and 0.078 V, respectively.

**Figure 4:**
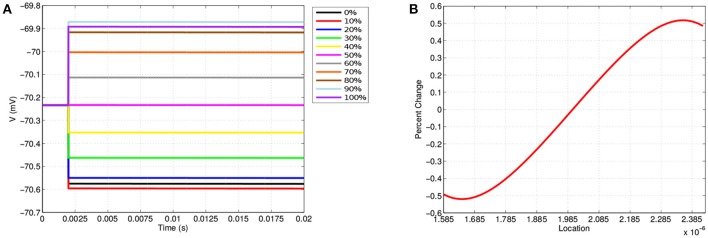
**(A)** Shows the transmembrane voltages due to TES application at equispaced locations within the node of Ranvier. **(B)** Shows the percent change in the transmembrane voltage due to TES at each point along the membrane; 1.585·10^-6^ μm is the far-left, 2·10^-6^ μm is the center, and 2.385·10^-6^ μm is the far right. A positive percent change indicates depolarization and a negative percent change indicates hyperpolarization.

Along the neuron membrane, there is a change in transmembrane voltage upon application of electrical stimulation after *t* = 2 ms (Figure 4). Figure 4A shows the transmembrane voltage throughout the simulation at 11 equispaced points within the node of Ranvier. These points are labeled as a percent based on their position along the node of Ranvier where, for example, 0, 50, and 100% refer to the points on the far left, middle, and far right of the node. The resting transmembrane voltage for each of these points is approximately -70.23 mV. For the point in the center of the node the transmembrane voltage does not change upon stimulation, maintaining its value of -70.23 mV throughout the simulation. For all other points, immediately at stimulation application, there is an instantaneous jump in transmembrane voltage, however, this change depends on the location along the membrane (Figure 4B).

These results demonstrate the location dependence of changes in transmembrane voltage due to TES. Specifically, transmembrane voltages at points left of center become hyperpolarized, whereas depolarization occurs on the right-hand side. In addition, the magnitude of the polarization from TES administration varies depending on proximity to the edges and center of the node of Ranvier; these values change to a greater degree near the edges as compared to locations near the center. Furthermore, Figure 4B shows that maximum changes in transmembrane voltage do not occur at the extreme edges of the node, but rather at locations situated at 1.64·10^-6^ μm and 2.35·10^-6^ μm, which correspond to approximately 9 and 91%, both well within the the edge of the node of Ranvier. Interestingly, hyperpolarization occurs for locations on the side with the 0.1 V stimulation source, whereas depolarization occurs on the side adjacent to the ground boundary condition.

In addition to these findings, it is observed that membrane voltage polarization is sustained throughout the TES application, which is consistent with clinical results that show that TES effects persist in sessions consisting of tens of minutes (Miniussi et al., 2008; Nitsche et al., 2008). This sustained increase in neural impulse sensitivity in specific regions of a node of Ranvier permits the TES treatment efficacy recognized by the medical field (Nitsche et al., 2008; Caparelli-Daquer et al., 2012). Our results are also consistent with clincal research that shows that TES has the net effect of increasing neuron excitability by depolarizing to sub-threshold potential (Liebetanz et al., 2002; Bikson et al., 2004; Stagg and Nitsche, 2011; Das et al., 2016). In addition, changes in transmembrane voltage magnitude are consistent with previous mathematical simulations of TES (Dougherty et al., 2014).

### 3.2. Voltage Gated Ion Channel State Variables Exhibit Location Specificity

**Figure 5:**
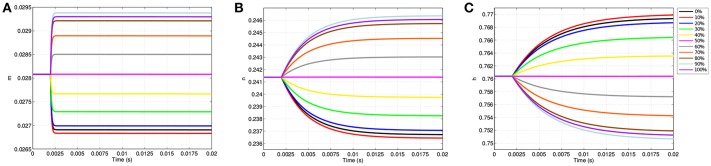
Gating variable values due to TES application at equispaced locations within the node of Ranvier. **(A)** Shows *m*, **(B)** shows *n*, and **(C)** shows *h*.

The changes in transmembrane voltage due to TES directly impact the behavior of voltage gated ion channels due to changes in their gating variables (Figure 5). Like Figure 4A, Figure 5 displays the values of each gating variable throughout the simulation at the same 11 equispaced points within the node of Ranvier. The location specificity previously observed with transmembrane voltage is also present for the changes in all gating variables.

Prior to stimulation application, *m, n*, and *h* show minimal position dependence as their respective values are essentially equal throughout the membrane. For example, before TES application, *m* is approximately 0.0281 everywhere in the node of Ranvier. When stimulation is applied, changes in *m, n*, and *h* become location specific; points where the cell becomes hyperpolarized, i.e., locations between 0 and 50%, result in decreases in *m* and *n* as well as increases in *h*. On the other hand, at sites of depolarization, namely positions between 50 and 100%, *m* and *n* increase while *h* decreases.

Directly corresponding to the locations of maximum change in transmembrane voltage, positions of greatest change in all gating variables also occur off of the membrane edges near 10 and 90%. In addition, the curves of the gating variables are directly associated to the polarized membrane voltages at the same 11 points. In particular, the amplitudes of the gating variable curves correspond to their associated transmembrane voltages, as well as distances between the curves; more precisely, the ranking of each curve of *m* based on plot amplitude is identical to the ranking of the transmembrane voltage curves, and in addition, the amount of spacing between *m* curves (Figure 5A) is proportional to the spacing between transmembrane voltage curves (Figure 4A). The same observations apply for *n* (Figure 5B), and *h* (Figure 5C) as well with the exception that the ordering is inverted due to characteristics of *h* that are subsequently discussed.

While the dependence of gating variables on transmembrane voltage is not unexpected, the location specificity of the gating variables due to TES shown here is novel, and in addition, begins to explain how neurostimulation impacts ion channel gating and subsequent ionic flux. Of particular interest in this regard, a clear difference in the shapes, magnitudes and trajectories of the *m, n*, and *h* time course curves is observable; the *m* gating variable changes rapidly, hitting a limiting value early in the simulation, whereas *n* and *h* grow more slowly, and fail to reach an asymptotic value within 20 ms. However, *m* has the lowest amplitude change of the three, with a maximum change of 0.0013, which is only 26.1 and 16.25% of the changes in *n* and *h*, respectively.

**Figure 6:**
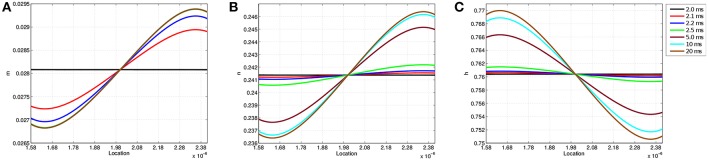
Gating variable values at each location along the node of Ranvier at simulation times *t* = 2.0, 2.1, 2.2, 2.5, 5, 10, and 20 ms. **(A)** Shows *m*, **(B)** shows *n*, and **(C)** shows *h*.

Figure 6 shows the values of each gating variable at every point along the discretized membrane at seven different simulation times. At *t* = 2 ms, each gating variable maintains the same value along the membrane as TES application has not yet started; after administration, the value of each gating variable changes over time based upon its location in the membrane. The speed at which *m* reaches its limiting value is also seen here as the curves for 5, 10, and 20 ms are virtually identical. In contrast, all curves for *n* and *h* are visible and continually change throughout the 20 ms simulation. Similar to transmembrane voltage, maximum and minimum values occur approximately at the 9 and 91% locations. Furthermore, it is seen that on the left-half of the node of Ranvier, the *m* and *n* probability values are lower than those attained on the right-half, and the opposite is true for *h*. As will be shown in section 3.3, the time and location dependence of changes in these gating variables as a result of TES has a direct impact on transmembrane ionic current.

### 3.3. Membrane Ion Flux Exhibits Location Specificity

**Figure 7:**
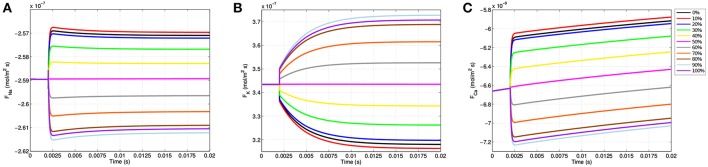
Membrane flux for sodium **(A)**, potassium **(B)**, and calcium **(C)** over the course of the simulations for the 11 equispaced points on the node of Ranvier. A negative flux indicates ion flow into the cell from the extracellular space, and a positive value indicates an efflux out of the cell.

As the gating variables dictate ion channel permeability, the location specificity observed in transmembrane voltage as well as *m, n*, and *h* has a direct influence on ion flux into and out of the neuron. Figure 7 shows the ion flux for sodium, potassium, and calcium over time at the 11 equispaced points within the node of Ranvier. Given the sign convention of the boundary condition governing membrane current (Equation 9), a negative value for flux indicates current coming into the cell from the extracellular space.

Due to passive electrodiffusion forces from the multi-ion environment, as well as a transmembrane voltage not precisely equal to -70 mV, a slight flux of ions across the membrane occurs prior to TES application. Upon activation after *t* = 2.0 ms, there are significant changes in neuronal flux. For locations on the right-half of the node of Ranvier, where the cell becomes depolarized (Figure 4B), there is an increase in sodium influx (Figure 7A). This is precisely predicted by the gating variable results (Figures 5A,C); as *m* represents sodium channel activation, which increases on the right-hand side, and *h*, sodium channel inactivation, which decreases on the right, an increase in sodium influx is this region is expected, and as shown in Figure 7A is attained. In addition, this influx is greatest at the 91% mark, which correlates with all prior results including (i) where the cell experiences its greatest depolarization, (ii) where *m* is maximal, and (iii) where *h* in minimal. On the hyperpolarized left-hand side, sodium influx still occurs, but at a decreased rate as *m* decreases and *h* increases here.

The *m* gating variable also controls calcium channel activation (see Appendix B), and so trends in calcium flux function similarly to sodium flux (Figure 7C). Specifically, locations where the cell becomes depolarized yield an increase in calcium influx and hyperpolarized regions experience a decreased influx. For potassium, due to its reversal potential, the opposite occurs and an efflux transpires throughout the entire node of Ranvier. In addition, as *n* governs potassium activation, potassium efflux increases on the left side where hyperpolarization presents and decreases on the right half of the node of Ranvier (Figure 7B).

These results are consistent with published TES studies that show an increase in calcium influx from a membrane depolarization due to an electric field applied in the extracellular medium (Nitsche et al., 2003; Adams et al., 2017). In addition, like the biological literature, our model predicts that this influx is governed by voltage gated calcium channel permeability (Islam et al., 1995). The novelty of this model is in extending this knowedge to provide a description of how the voltage gated calcium channels within the node of Ranvier operate to achieve this. First, the model allows to see the changes in flux at a greater frequency and with more spacial detail than has been captured with experiments. In addition, the model identifies the gating variable *m* as driving the changes in flux. Finally, these results reveal a time and spatial based dependence of the gating variable, voltage gated channel activation, and calcium flux.

### 3.4. TES Causes Intracellular Calcium Dyshomeostasis

**Figure 8:**
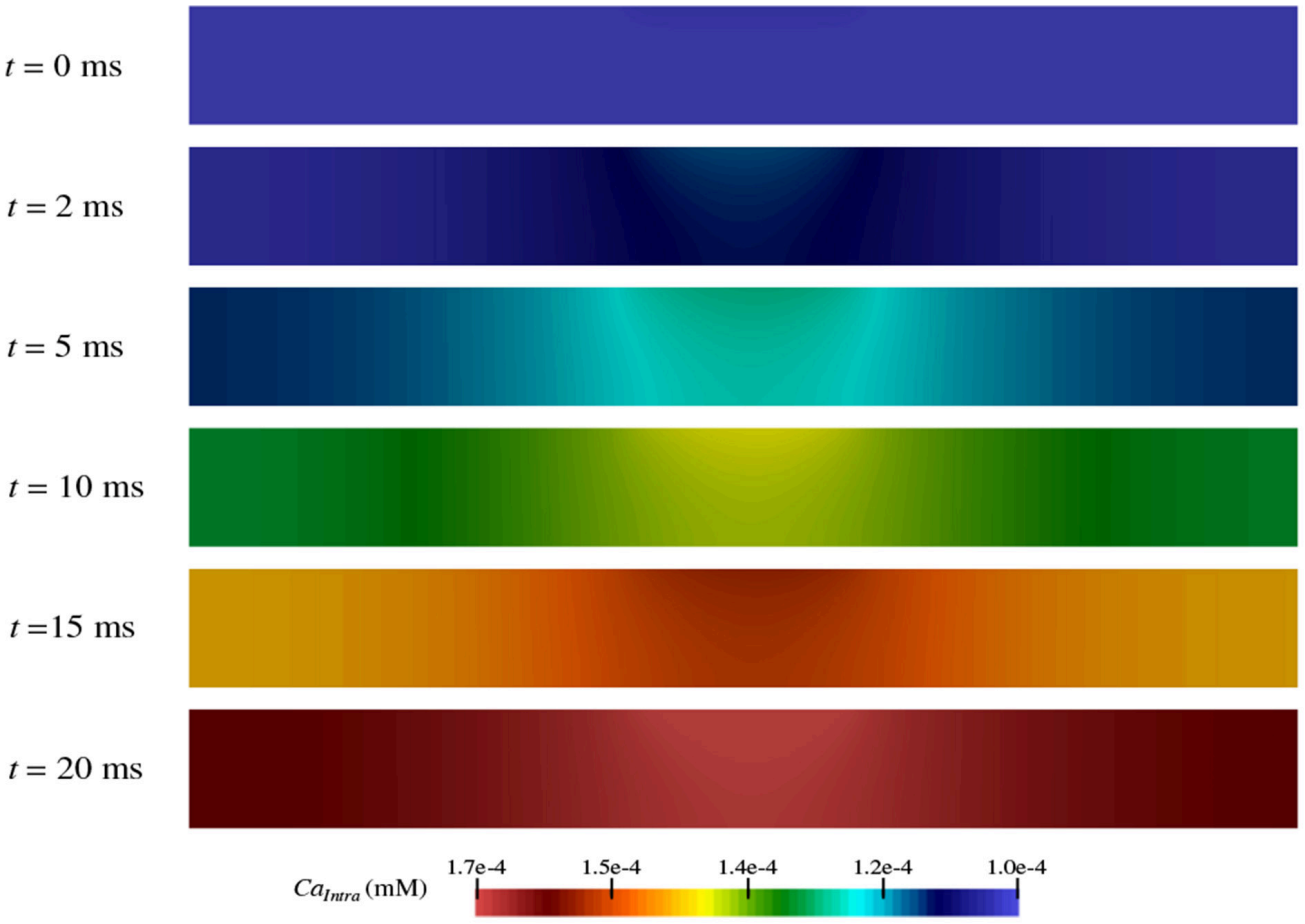
Concentration of calcium in the intracellular space (Ω_*I*_ in Figure 1) during the simulation at time *t* = 0, 2, 5, 10, 15, and 20 ms.

As shown in section 3.3, calcium flows into the neuron from the extracellular space at different rates depending on the region within the node of Ranvier (Figure 7C). Thus, over the course of the TES simulation, an increase in intracellular calcium concentration occurs. However, the magnitude and rate of this increase is unknown. Figure 8 shows intracellular calcium concentrations at six simulation time steps. At *t* = 0 ms, the entire intracellular space has a constant concentration of 10^-4^ mM, which is the initial condition for calcium in this domain. Over time, an increase in calcium concentrations from calcium flux due to TES is seen at all subsequent time steps. In addition, for times *t* > 0, a larger concentration of calcium is noticed at the membrane region, precisely due to calcium influx at the membrane, along with a diffusion throughout the intracellular domain. At the 91% membrane location calcium concentrations increase by 71.65% over the course of the simulation. Furthermore, the total amount of calcium within the intracellular space increases by 63.86% during the course of the simulation. This increase is approximately linear, as can be seen from the color gradients of the intracellular concentration plots.

These results are consistent with prior experiments that found an increase in calcium concentration due to an influx of calcium in the presence of electrical stimulation (Islam et al., 1995; Adams et al., 2017). In fact, the values predicted by the model are within one order of magnitude of those shown in electrical stimulation biological studies (Adams et al., 2017). Moreover, the model augments this knowledge by provding a detailed prediction of how, where, and when calcium ion flow into the neuron as described in section 3.3.

## 4. Discussion

Mathematical modeling and computer-based simulation has shown to be a valuable component in enhancing neurostimulation efficacy as well as providing an instrument for helping the research community learn about the mechanisms by which it operates. While both *in silico* and biological experimentation have facilitated a greater understanding of neuromodulation, the cellular-level electrodynamics during electrical stimulation treatments still remain highly elusive. To help address this contention, we have presented a novel mathematical model of transcranial electrical stimulation that describes the effect of TES on ion channel dynamics and transmembrane ionic flux. The model is based on the Poisson-Nernst-Planck system of partial differential equations, and to our knowledge is the first that integrates electric potential energy, individualized ion species, voltage-gated ion channels, and transmembrane flux with a medically-based TES induced electric field, and within a biologically-inspired computational domain, showcases how this treatment effects neuronal electrical functioning.

A key finding of this work is the location specificity exhibited by the cell's electrical processes due to TES. In particular, results show that TES polarizes the neuron as expected, however, the degree of voltage change is dependent on the location within a node of Ranvier, a phenomena reported by the deep brain stimulation modeling community (McIntyre et al., 2003, 2004). In turn, results show that the states of the ion channels also exhibit location-dependent changes, which directly impacts membrane flux and subsequent intracellular sodium, potassium, calcium, and chloride concentrations. While the degree and type of electrical polarization is location dependent, these results show that TES effectively elevates resting membrane potential so that ultimately neuron firing is more achievable (Nitsche et al., 2008).

It is well-known that cytosolic calcium is a key element in the intracellular signaling cascade that enables neurotransmitter secretion as well as cell viability. In addition, a disruption to calcium homeostasis is correlated with neurodegenerative disease (Bezprozvanny, 2009; Marambaud et al., 2009; Calì et al., 2014; Surmeier et al., 2017). Our results augment these findings by showing that TES directly alters calcium membrane flux and intracellular calcium concentration via voltage gated calcium channels, by almost 64% over the course of the simulation. These findings may suggest that a possible mechanism by which neurostimulation achieves therapeutic success, in addition to depolarizing the cell, is by altering calcium dyshomeostasis in diseased neurons.

**Figure 9:**
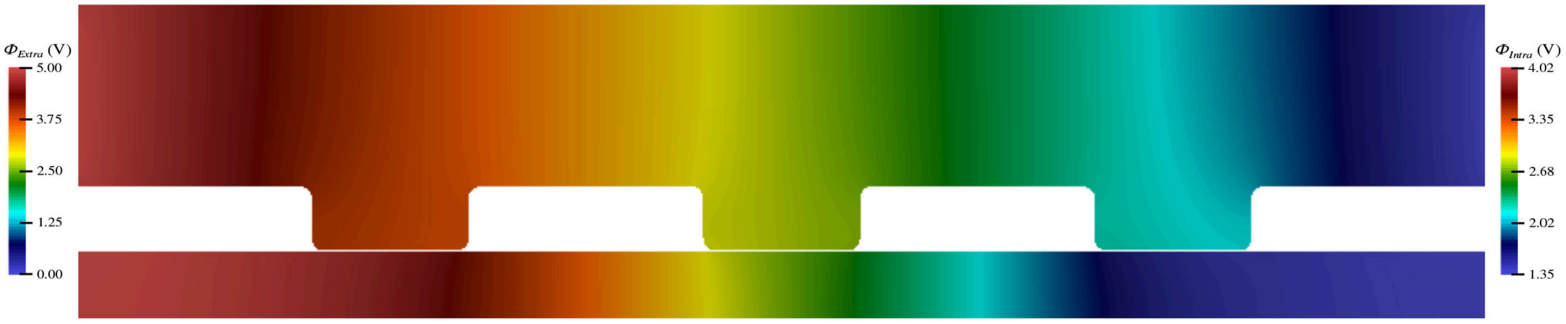
Electric potential energy due to TES throughout a computational domain of a neuron with three nodes of Ranvier.

**Figure 10:**
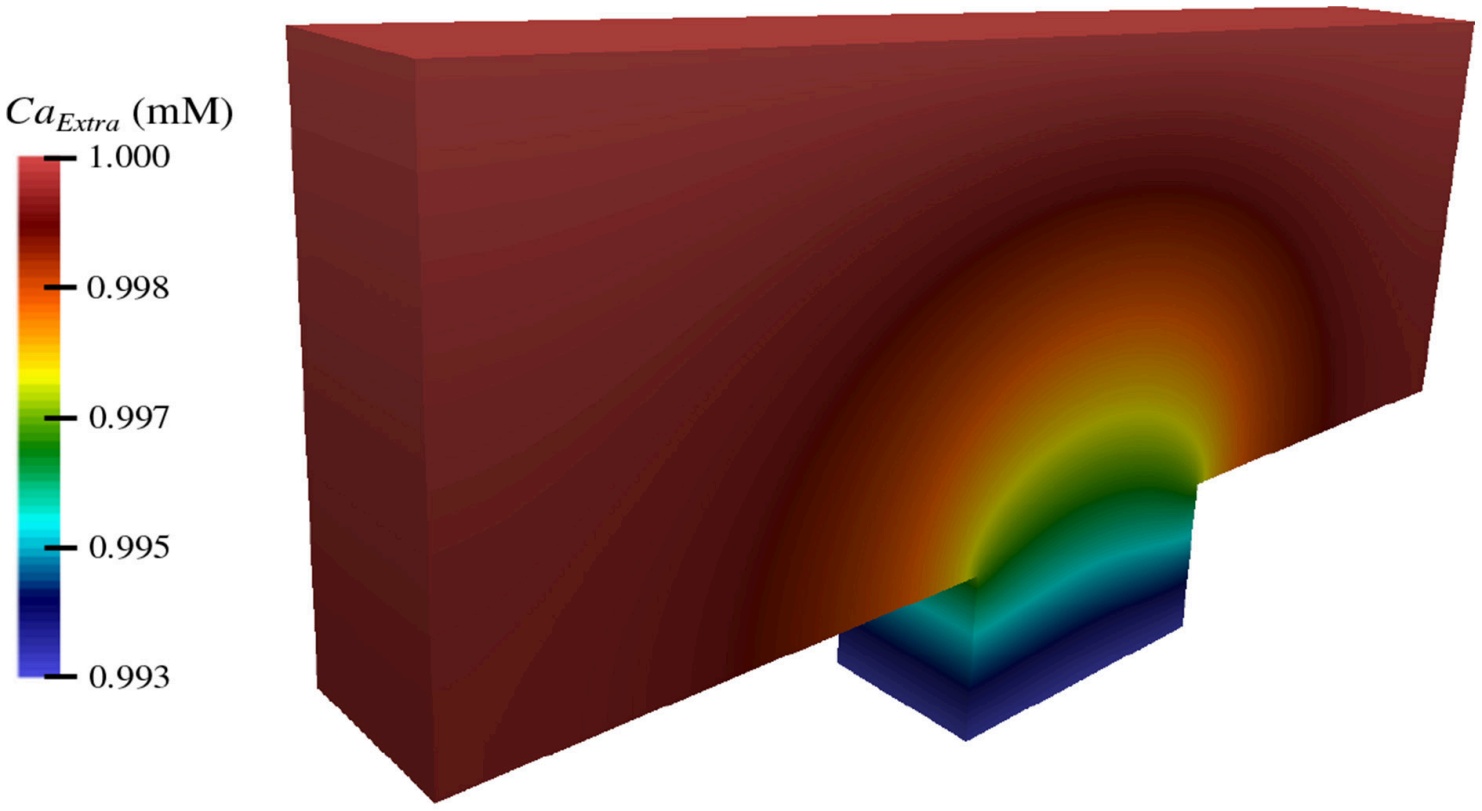
Extracellular concentration of calcium during TES in a three-dimensional computational domain.

By implementing the simulation software using an object-oriented approach, its utility can be seamlessly extended to other computational studies and future work. Using these tools, we have begun investigating the impact of TES on more biologically complex domains, including one that encompasses three nodes of Ranvier (Figure 9). In addition, we are starting to examine the effect of TES on three-dimensional domains (Figure 10). We have also begun to compare the influence of different forms of neurostimulation, like deep brain stimulation, on transmembrane ionic flux. Finally, we are interested in examining the effects of ionic flux and cytosolic ion concentrations on intracellular signaling pathways that have implications to neurodegenerative disorders.

## Data Availability

The raw data supporting the conclusions of this manuscript will be made available by the authors, without undue reservation, to any qualified researcher.

## Author Contributions

All authors listed have made a substantial, direct and intellectual contribution to the work, and approved it for publication.

### Conflict of Interest Statement

The authors declare that the research was conducted in the absence of any commercial or financial relationships that could be construed as a potential conflict of interest.
