# Supplementary material for: Location Specificity of Transcranial Electrical Stimulation on Neuronal Electrodynamics: A Mathematical Model of Ion Channel Gating Dynamics and Ionic Flux Due to Neurostimulation
Source: Front Comput Neurosci. 2019 Apr 4;13:17. doi: 10.3389/fncom.2019.00017 (PMC6458477; doi:10.3389/fncom.2019.00017)
Supplement: Supplementary file 1 [file Data_Sheet_1.pdf]

Appendix A PDE Weak Formulation Derivations

The derivations of the weak formulations for the Poisson and Nernst-Planck equations for the finite element method are shown here.

Poisson Equation

$$\text{Let } f = -F\sum_{i=1}^{n} z_i n_i$$

Given $f \,\epsilon\, L^2(\Omega)$, multiply equation 3 by an arbitrary test function, v, and integrate over the domain Ω:

$$\int_\Omega \nabla\cdot(\epsilon\nabla\phi)v\; d\Omega = \int_\Omega f(n)v\; d\Omega.$$

Applying Green's Theorem yields:

$$\int_\Omega \epsilon\nabla\phi\cdot\nabla v\; d\Omega = -\int_\Omega f(n)v\; d\Omega + \int_\Gamma v(\vec{\boldsymbol{n}}\cdot\epsilon\nabla\phi)\; ds$$

Applying boundary conditions given by equation 8 gives:

$$\int_\Omega \epsilon\nabla\phi\cdot\nabla v\; d\Omega = -\int_\Omega f(n)v\; d\Omega.$$

We stipulate the solution space to inforce the Dirichlet boundary conditions in equations 5 and 6, and arrive at the weak formulation: Given $f \,\epsilon\, L^2(\Omega)$, find $\phi \,\epsilon\, H_0^1(\Omega)$ such that:

$$\int_\Omega \epsilon\nabla\phi\cdot\nabla v\; d\Omega = -\int_\Omega f(n)v\; d\Omega,\ \text{for all } v \,\epsilon\, H_0^1(\Omega),$$

where $H_0^1(\Omega) = \{u \mid u \,\epsilon\, H^1(\Omega) \text{ and } u = 0 \text{ on } \Gamma_1\}$.

Nernst-Planck Equation

Discretize the time derivative in equation 1 using the θ-rule:

$$\frac{\partial n_i}{\partial t} \approx \frac{n_i^{k+1} - n_i^k}{\Delta t} = \theta(-\nabla\cdot F_i^{k+1}) + (1-\theta)(-\nabla\cdot F_i^k)$$

Given $F_i \,\epsilon\, L^2(\Omega)$, multiply equation 1 by an arbitrary test function v and integrate over Ω:

$$\int_\Omega n_i^{k+1}v\; d\Omega = \int_\Omega n_i^k v\; d\Omega - \theta dt\int_\Omega(\nabla\cdot F_i^{k+1})v\; d\Omega - dt(1-\theta)\int_\Omega(\nabla\cdot F_i^k)v\; d\Omega.$$

Applying Green's Theorem yields:

$$\begin{aligned}\int_\Omega n_i^{k+1}v\; d\Omega = &\int_\Omega n_i^k v\; d\Omega - \theta dt\int_\Gamma(F_i^{k+1}\cdot\vec{\boldsymbol{n}})v\; ds + \theta dt\int_\Omega F_i^{k+1}\cdot\nabla v\; d\Omega \\ &- dt(1-\theta)\int_\Gamma(F_i^k\cdot\vec{\boldsymbol{n}})v\; ds + dt(1-\theta)\int_\Omega F_i^k\cdot\nabla v\; d\Omega.\end{aligned}$$

Applying boundary conditions given by equations 7 and 9 gives:

$$\int_{\Omega} n_i^{k+1} v \, d\Omega - \theta dt \int_{\Omega} F_i^{k+1} \cdot \nabla v \, d\Omega = \int_{\Omega} n_i^{k} v \, d\Omega - \theta dt \int_{\Gamma_4} f_i^{memb^{k+1}} v \, ds$$
$$- dt(1-\theta) \int_{\Gamma_4} f_i^{memb^{k}} v \, ds + dt(1-\theta) \int_{\Omega} F_i^{k} \cdot \nabla v \, d\Omega.$$

We stipulate the solution space to enforce the Dirichlet boundary conditions in equation 4 and arrive at the weak formulation: Given $F_i \,\epsilon\, L^2(\Omega)$, find $n^{k+1} \,\epsilon\, H^1_{n_i}(\Omega)$ such that:

$$\int_{\Omega} n_i^{k+1} v \, d\Omega - \theta dt \int_{\Omega} F_i^{k+1} \cdot \nabla v \, d\Omega = \int_{\Omega} n_i^{k} v \, d\Omega - \theta dt \int_{\Gamma_4} f_i^{memb^{k+1}} v \, ds$$
$$- dt(1-\theta) \int_{\Gamma_4} f_i^{memb^{k}} v \, ds + dt(1-\theta) \int_{\Omega} F_i^{k} \cdot \nabla v \, d\Omega,$$
$$\text{for all } v \,\epsilon\, H^1_{n_i}(\Omega).$$

Appendix B Hodgkin-Huxley Gating Equations

To calculate the transmembrane flux for each ion, a Hodgkin-Huxley scheme is used (Hodgkin et al., 1952). This model was adapted from prior PNP simulations (Dione et al., 2016) by including chloride and calcium current and conductances (Tuckwell, 2012; Kay et al., 1987).

The model includes ordinary differential equations describing the kinetics of the gating variables m, n, and h. These equations depend on the transmembrane voltage, given by $V = \phi_I$ - ϕ_E. These gating variables are probabilities between 0 and 1 that indicate ion channel state, controlling the opening and closing of the voltage gated channels. This system of ODEs is given by:

$$\frac{dn}{dt} = \alpha_n(V)(1-n) - \beta_n(V)n, \tag{10}$$

$$\frac{dm}{dt} = \alpha_m(V)(1-m) - \beta_m(V)m, \tag{11}$$

$$\frac{dh}{dt} = \alpha_h(V)(1-h) - \beta_h(V)h, \tag{12}$$

where α and β are prescribed functions of the transmembrane voltage (Dione et al., 2016).

These gating variables are used in the calculation of the membrane current for each ion, given by:

$$I_{Na^+} = (g^v_{Na} m^3 h + g^l_{Na}) \cdot (V - E_{Na}), \tag{13}$$

$$I_{K^+} = (g^v_K n^4 + g^l_K) \cdot (V - E_K), \tag{14}$$

$$I_{Ca^{+2}} = (g^v_{Ca} m^2 + g^l_{Ca}) \cdot (V - E_{Ca}), \tag{15}$$

$$I_{Cl^-} = (g^v_{Cl} + g^l_{Cl}) \cdot (V - E_{Cl}), \tag{16}$$

where E_i represents the Nernst potential of each ion and is given by:

$$E_i = \frac{RT}{z_i F} ln\left(\frac{n_i^{extra}}{n_i^{intra}}\right), \tag{17}$$

and g^l_i represents the conductance of leak channels whereas g^v_i is the conductance of the voltage gated channel for each ion. These conductance values are shown in Table 2.

Table 2: Ion Channel Conductance Parameters

Parameter	Value
g^v_{Na}	1000.0 S/m^2
g^v_K	400.0 S/m^2
g^v_{Ca}	9.0 S/m^2
g^v_{Cl}	0.2 S/m^2
g^l_{Na}	0.175 S/m^2
g^l_K	0.50 S/m^2
g^l_{Ca}	0 S/m^2
g^l_{Cl}	0 S/m^2

These equations are used to calculate the flux for each ion, given by:

$$f_i^{memb} = \frac{I_i}{z_i F}, \tag{18}$$

where z_i is the valence of ion i and F is Faraday's constant.
